# [Corrigendum] Inhibition of human oral squamous cell carcinoma proliferation and migration by prodrug‑activating suicide gene therapies

**DOI:** 10.3892/etm.2023.12159

**Published:** 2023-08-07

**Authors:** Naining Xu, Honglei Tian, Chun Po Fung, Yuntao Lin, Yuling Chen, Guang Zhu, Yuehong Shen, Chuanbin Guo, Hongyu Yang

Exp Ther Med 25:92, 2023; DOI: 10.3892/etm.2023.11790

Subsequently to the publication of the above article, the authors have realized that, for the scratch-wound healing assay experiments shown on p. 6 and 7, the same image had inadvertently been selected to show the results of the ‘24 h observation’ experiments presented in Figs. 5A and 6A. After having consulted their original data, the authors have realized that Fig. 5A was assembled incorrectly. The revised version of Fig. 5, now showing the correct data for the ‘24 h observation’ experiment in Fig. 5A, is shown on the next page. Note that the error made during the assembly of this figure did not grossly affect either the results or the conclusions reported in this study, and all the authors agree with the publication of this corrigendum. Furthermore, they are grateful to the Editor of *Experimental and Therapeutic Medicine* for allowing them to publish this, and apologize to the readership for any inconvenience caused.

## Figures and Tables

**Figure 5:**
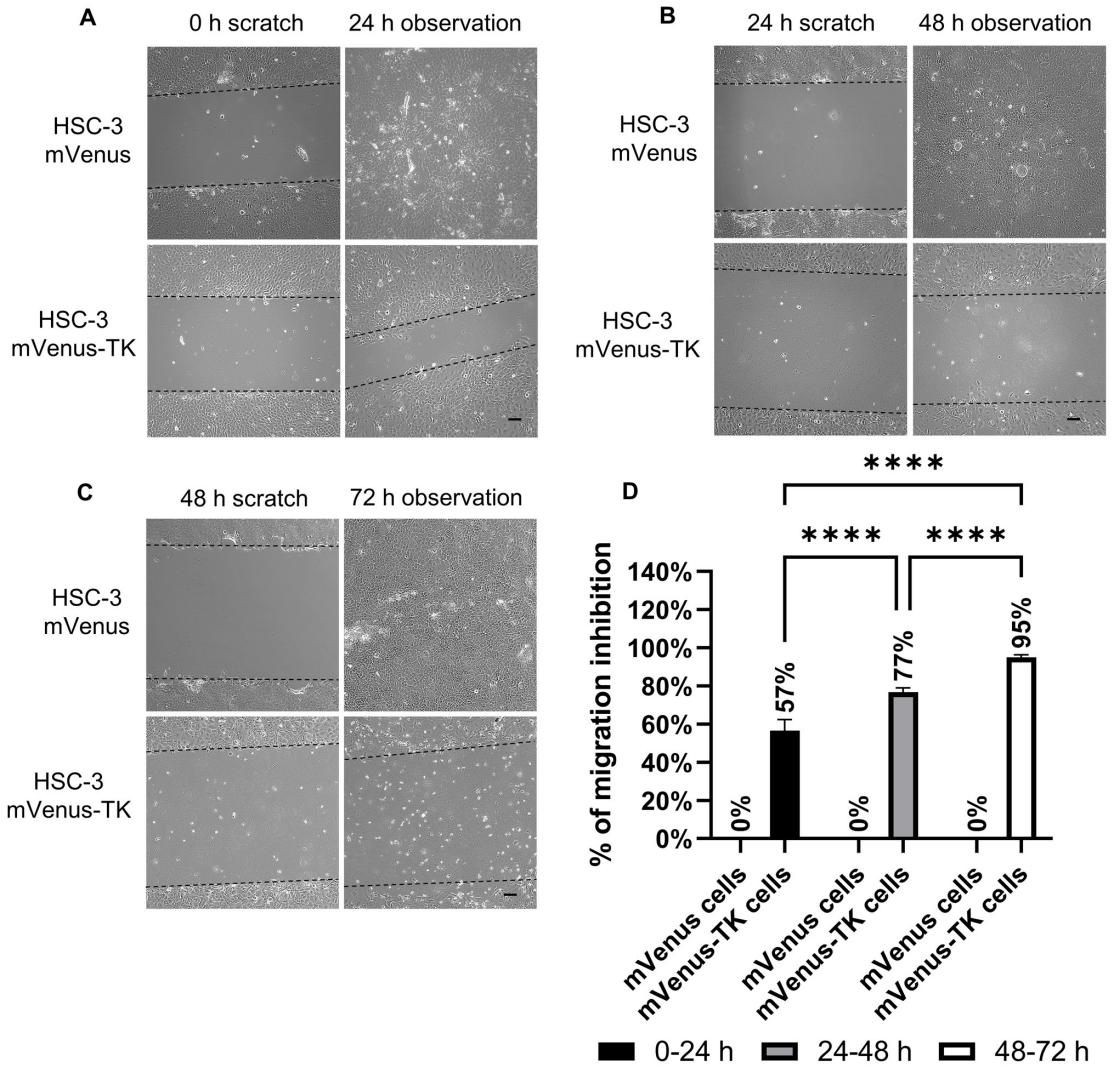
Inhibition of HSC-3 mVenus-TK cell migration following treatment with GCV. (A) GCV treatment inhibited HSC-3 mVenus-TK but not HSC-3 mVenus cell migration during the 0-24 h period. Following generation of the wound, scratch images were captured and cells were treated with 25 µM GCV. The healing images were captured after a further 24 h GCV treatment (scale bar, 100 µm). (B) GCV treatment inhibited HSC-3 mVenus-TK cell migration during the 24-48 h period. HSC-3 mVenus and HSC-3 mVenus-TK cells were treated with 25 µM GCV for 24 h and wounds were generated. After taking the scratch images, cells were treated with 25 µM GCV for a further 24 h, then the healing images were captured (scale bar, 100 µm). (C) GCV treatment inhibited HSC-3 mVenus-TK cell migration during the 48-72 h period. The wounds were generated after cells were treated with 25 µM GCV for 48 h. The cells were treated for a further 24 h, then healing images were captured. Scale bar, 100 µm. (D) Quantification of inhibition of cell migration for treatment durations. The percentage of migration inhibition is the ratio of the area of healing images to the average strip area of the scratch images. Data are presented as the mean ± SD of >3 healing images. Data were analysed using one-way ANOVA followed by Tukey’s post hoc test for multiple comparisons. ^****^P<0.0001. CD, cytosine deaminase; TK, thymidine kinase; GCV, ganciclovir; SD, standard deviation.

